# Internal Exposure Levels and Health Risk Assessment of Melamine and Organophosphate Metabolites in Urine: Research Progress and Prospects

**DOI:** 10.3390/toxics13110950

**Published:** 2025-11-04

**Authors:** Qu Zhang, Qi Jiang, Xin-Hong Wang, Liang Wang, Mei-Hua Tian, Da-Zhong Chen, Chun-Yan Huo, Wen-Long Li

**Affiliations:** 1 College of the Environment and Ecology, Xiamen University, Xiamen 361102, China; 2 Third Institute of Oceanography, Ministry of Natural Resources, Xiamen 361005, China

**Keywords:** biomonitoring, human exposure, emerging pollutants, metabolites, human health

## Abstract

With the widespread use of emerging contaminants such as melamine (MEL) and organophosphate esters (OPEs) as alternatives to traditional flame retardants, their ubiquitous presence in the environment has raised concerns about human internal exposure and health risks. Urine, as a critical matrix for biomonitoring, enables accurate assessment of internal exposure to these contaminants and their metabolites. This review systematically summarizes the research progress on urinary biomonitoring of MEL and its derivatives (cyanuric acid (CYA), ammeline (AMN), ammelide (AMD)) and OPE metabolites. It covers analytical methods (sample pretreatment including enzymatic hydrolysis and extraction, instrumental detection via HPLC-MS/MS/UPLC-MS/MS, and method validation), exposure characteristics (global spatial differences, population disparities among sensitive groups like children and e-waste workers, and temporal variations such as postprandial peaks), and health risk assessments. Results show that MEL and CYA are widely detected in urine (detection rates > 97%), with CYA dominating total MEL (66.2–80%); OPE metabolites exhibit regional compositional differences, e.g., bis(2-chloroethyl) phosphate (BCEP) in Shenzhen and diphenyl phosphate (DPHP) in New York. Current exposure levels are generally safe, but 2–12% of sensitive individuals face potential risks. This review highlights key challenges (method standardization, limited hydroxylated OPE standards) and provides directions for future research to establish a comprehensive exposure–health risk evaluation system.

## 1. Introduction

With the acceleration of industrialization and rising consumption levels, large quantities of chemicals (e.g., plasticizers, flame retardants, preservatives, ultraviolet filters) have brought convenience to daily life while simultaneously causing severe environmental pollution issues. Human exposure to these substances is becoming increasingly widespread. Currently, approximately 150,000 chemicals are registered under the European Union’s Registration, Evaluation, Authorisation and Restriction of Chemicals (REACH) regulatory program [1]. Traditional flame retardants such as polybrominated diphenyl ethers (PBDEs) are banned due to their persistence, bioaccumulative toxicity, and potential for long-range transport [2]. As alternatives to PBDEs, melamine (MEL) and organophosphate ester (OPE) flame retardants have been used in a wide range of products, including coatings, plastics, furniture, building materials, and electronic equipment [3,4,5].

Environmental sources of melamine exposure also include everyday food, house dust, tableware, clothing, and decorative coatings [6,7,8,9,10]. The main derivative, cyanuric acid (CYA), is commonly used in disinfectants, bleach, and fertilizers [10]. Melamine diamide (AMD) and melamine monoamide (AMN), on the other hand, are intermediates produced during the hydrolysis of melamine and are present as impurities in melamine preparations (Table 1). MEL and its derivatives are widely present in various environmental media, such as food, indoor dust, and drinking water. Indoor dust MEL concentrations are higher in China and the United States, but 2–3 orders of magnitude lower than the exposure dose from dietary intake [9]. Concentrations of MEL and CYA in U.S. drinking water are much higher than in bottled water, and CYA is abundant in swimming pools, a major source of CYA exposure [11].

OPEs are also frequently used as plasticizers and lubricants in items like children’s products and cosmetics [12,13]. Notably, OPEs are physically rather than chemically bound to the matrices of products. This characteristic enables them to easily leach out from products and infiltrate the surrounding environment during usage, resulting in their widespread presence in diverse environmental media (e.g., soil, surface water, sediments, agricultural products, and dust) [14,15,16]. Indoor dust in China shows relatively high levels of OPEs, ranging from 61.4 to 9,694,000 μg/g, primarily due to the widespread use of OPE-containing construction and decorative materials [17,18]. Additionally, OPEs have been reported in food and drinking water in China, with rice identified as a major source of OPE exposure [19].

MEL and its derivatives have a major impact on kidney health, as MEL forms MEL-URATE crystals with urate, and subsequent studies have shown that MEL-CYA crystals are formed when MEL and CYA are ingested at the same time, which can lead to the formation of stones in the kidneys and thus cause kidney damage [20,21]. In recent years, the French Agency for Food, Environment, and Occupational Health and Safety (ANSES) has classified melamine as an endocrine disruptor [22]. Many studies have confirmed that melamine causes endocrine disruption, kidney toxicity, neurotoxicity, and other problems [21,23,24]. Similarly, a growing number of studies have categorized OPEs as endocrine-disrupting chemicals (EDCs). Exposure to triphenyl phosphate (TPHP) and tris (1,3-dichloro-2-propyl) phosphate (TDCIPP) disrupts the secretion of thyroid hormones and sex hormones in zebrafish and in vitro models [12,25]. In addition, tris (2-chloroethyl) phosphate (TCEP), tri-n-butyl phosphate (TNBP), and triphenyl phosphate are neurotoxic [26].

External and internal exposure assessments can both be used to evaluate human exposure to chemicals and associated health risks [6,27,28]. Among the various assessment methods, biomonitoring is the most relevant for assessing the health impacts of human internal exposures [29]. The main samples for biomonitoring include blood, human milk, lipids, urine, hair, and other matrices, with urine serving as a critical matrix for biomonitoring [30]. The majority of metabolites produced after the human body processes these compounds are excreted via urine. The highest concentrations of MEL have been reported to be detected in the kidneys and bladder, with high plasma clearance, and more than 90% of MEL and CYA are excreted in the urine [22,31]. MEL and CYA were the main biomarkers, with a detection rate of more than 97% in the urine of the general population in 13 cities in China, with CYA accounting for 66.2% of ∑MEL [32]. The metabolism of OPE in the human body is incomplete, but it is known that most OPE is metabolized to diester metabolites. Common urinary OPE biomarkers include di-n-butyl phosphate (DNBP), di-iso-butyl phosphate (DIBP), diphenyl phosphate (DPHP), and bis(1,3-dichloro-2-propyl) phosphate (BDCIPP) (Table 1). Given the widespread exposure of the general population to OPEs, the detection rate of metabolites such as DPhP in urine exceeds 95% [33]. These hydrophilic metabolites are readily excreted in urine, making urinary metabolites a reliable biomarker of OPE exposure [34,35]. Consequently, urinary metabolites act as biomarkers reflecting the impact of the parent compounds on the human body and are used to assess internal exposure levels [12,36,37,38,39,40].

Given the widespread exposure to alternative flame retardants (MEL and its derivatives as well as OPEs), it is necessary to review the literature on human internal exposure to these chemicals and evaluate current detection methods, concentrations in urine, and health risk assessments, thereby proposing directions for future research. Current literature on urinary exposure to MEL, its derivatives, and OPE metabolites remains limited. This review discusses the advantages and limitations of current analytical methods, the concentration distribution patterns of these chemicals, and the assessed health risks associated with their exposure.

## 2. Methodology

A systematic literature search was conducted in Web of Science, ACS Publications, Springer, and PubMed using the keywords “melamine”, “cyanuric acid”, “organophosphate ester metabolites”, “urine”, “health risks”, and “risk assessment”. Studies published between 2000 and September 2025 were included, yielding approximately 14,730 search results. Literature was selected based on the following inclusion criteria: (1) studies published in mainstream scientific journals; (2) studies reporting the fate, occurrence, ecological risks, exposure levels, and health risk assessments of MEL and its derivatives, as well as OPEs metabolites in urine; (3) studies describing sample pretreatment and instrumental detection methods for MEL and its derivatives, and OPEs metabolites; (4) human urine studies conducted in different countries and regions. Following title and abstract screening, supplemented by manual review and full-text evaluation of the retrieved results, approximately 97 papers were retained. These papers focused on detection methods, urinary concentrations, internal exposure levels, and health risk assessments of MEL and its derivatives (MELs) and OPEs metabolites in urine.

The impacts of sample pretreatment and instrumental methods on target recovery rates, limits of detection/quantification (LOD/LOQ), and concentration levels were summarized. Additionally, the concentration levels, exposure levels, and health risks of target analytes in urine from different populations across various regions were synthesized. Statistical analyses were performed using Excel, and data visualization and further analyses were conducted using Origin 2024. Concentration levels were uniformly expressed in ng/mL, estimated daily intakes (EDI) in ng/kg bw/day, and tolerable daily intakes (TDI) in μg/kg bw/day.

## 3. Analytical Methods for Target Chemicals in Urine

### 3.1. Sample Preparation

Sample preparation is a critical step in the analysis of biological samples. Urine contains high levels of interfering matrices and endogenous compounds, such as proteins and lipids, which can induce significant matrix effects. It is therefore necessary to remove these impurities before instrumental analysis [29]. On one hand, particulate salts present in urine may cause clogging of the analytical instrument tubing. Many environmental pollutants undergo metabolic transformations in the human body—such as oxidation, hydrolysis, hydroxylation, sulfation, and alkylation—after exposure. The resulting metabolites may further undergo Phase II conjugation reactions with glucuronic acid or other biomolecules to form conjugated metabolites [41]. Previous reports indicate that Phase II conjugates bound in the form of glucuronic acid and sulfate are generally excreted via urine, including OPEs, polycyclic aromatic hydrocarbons (PAHs), environmental phenols (EP), organophosphorus (OP), and pyrethroid compounds [42]. These compounds can interfere with the lock mass calibration during high-resolution mass spectrometry (HRMS) analysis, thereby compromising the identification of biomarkers for target chemical exposures [43,44]. Therefore, enzymatic hydrolysis is required to eliminate endogenous compounds, along with sample extraction and purification, to ensure the stability and reliability of instrumental analysis.

#### 3.1.1. Enzymatic Hydrolysis

Uridine 5′-diphospho-glucuronosyltransferases (UGTs) in humans play a crucial role in the metabolic fate of numerous compounds. These enzymes catalyze the conjugation of glucuronic acid to substrates containing sulfhydryl, hydroxyl, aromatic amino, or carboxylic acid moieties. The resulting glucuronide conjugates exhibit significantly higher polarity than their parent compounds and are typically excreted from the body via renal pathways.

In the pretreatment of urine samples, the addition of β-glucuronidase or a β-glucuronidase/arylsulfatase enzyme mixture is a common and critical step, aiming to convert conjugated metabolites (formed by the binding of target compound metabolites to biomolecules such as glucuronic acid or sulfate groups) into free-form molecules. For instance, OPE metabolites are primarily excreted in urine as a mixture of glucuronide and sulfate conjugates. These metabolic conjugates require enzymatic hydrolysis by arylsulfatase (which can be used in combination with β-glucuronidase) to ensure that the detection results truly reflect the total exposure levels of target compounds in samples, thereby avoiding the underestimation of exposure levels caused by uneluted or undetected conjugated metabolites [42,45]. Consequently, the enzymatic hydrolysis step serves as a core prerequisite for recovery validation.

Owing to the structural stability of melamine and its derivatives, coupled with the presence of a triazine ring in their molecular structure, it is generally accepted that mammals are unable to metabolize these compounds [46]. Studies have shown that 90% of melamine is excreted via urine within 24 h [20]. Consequently, such chemicals do not form metabolic conjugates with glucuronic acid; thus, there is no need to add β-glucuronidase/arylsulfatase for enzymatic hydrolysis during the sample pretreatment process [3,8,27,37,47].

For OPE metabolites, β-Glucuronidase/Sulfatase Complex Enzyme Preparation is a commonly used hydrolase (Table S2). Hoffman et al. explicitly selected a complex enzyme containing β-glucuronidase (1000 U/mL) and sulfatase (33 U/mL) when analyzing pregnant women’s urine. The enzyme solution was prepared in 0.2 M sodium acetate buffer to ensure complete hydrolysis of conjugated metabolites [48]. Hammel et al. employed a complex enzyme with identical activity parameters for infant urine studies, followed by solid-phase extraction (SPE)–liquid chromatography–tandem mass spectrometry (LC–MS/MS) detection [49]. Yang et al. enhanced detection sensitivity in the HOME cohort study by adding the complex enzyme to efficiently hydrolyze conjugated OPE metabolites [50].

Regarding buffers, Hoffman et al. added 1.75 mL of 1 M sodium acetate buffer (pH 5) to 5 mL urine samples to provide a stable environment for enzyme activity [48]. Hammel et al. directly used 10 mM ammonium acetate buffer (pH 5.0), simplifying the workflow while ensuring efficient enzymatic hydrolysis [49]. Zhang et al., Hernandez-Castro et al., and Yang et al. exhibited slight variations only in buffer concentration (0.1–1 M), but all maintained a final pH of 5.0 to maximize enzyme activity [26,50,51].

The incubation temperature was uniformly set at 37 °C. Hoffman, Hammel, and Yang employed overnight incubation (12–16 h) to ensure hydrolysis efficiency [48,49,50]. Hernandez-Castro et al. reduced incubation to 4–6 h when processing 0.5 mL urine samples [51]. Zhang et al., working with 10 mL samples, extended incubation to 18 h to prevent incomplete hydrolysis [26].

#### 3.1.2. Extraction and Enrichment

##### MEL and Its Derivatives

As the core matrix for the biomonitoring of MEL and its derivatives, urine requires extraction and enrichment methods that are compatible with both the acid–base properties of target compounds (alkaline for MEL; acidic for CYA/AMD) and the characteristics of urine matrices (containing creatinine, organic acids, and water-soluble impurities) (Table S1). Currently, LLE dominates as the mainstream method due to its simplicity and low cost, while SPE is only used as an auxiliary technique in a few complex scenarios. These methods can be specifically categorized into two strategies: simultaneous extraction of multiple components and sequential extraction of single components, which exhibit significant differences in pH adjustment, solvent selection, and method performance [3,8,27,37,47].

The fundamental principle of LLE lies in achieving selective partitioning of target compounds through pH adjustment. The two-step LLE for simultaneous extraction of multiple components is suitable for the combined detection of multiple MEL derivatives. Zhu et al. took 250 μL of urine, split it into two equal aliquots, and treated each aliquot separately: one aliquot was basified with 5% ammonium hydroxide (NH_4_OH) for the extraction of MEL and AMN, while the other was acidified with 1% formic acid (FA) for the extraction of CYA and AMD. Ethyl acetate/isopropanol (EtAc/IPA, v/v = 95/5) was used as the extraction solvent for two consecutive cycles, resulting in recoveries ranging from 69% to 94% and a relative standard deviation (RSD) of <12% [37]. Liu et al. optimized this method by using pure EtAc as the extraction solvent to reduce matrix interference, thereby increasing recoveries to 72–88% [27]. A subsequent modification simplified the process to “one-step LLE with pH gradient adjustment” [47].

In contrast, the “sequential LLE for separate extraction of single components” is designed for the targeted detection of individual compounds. For MEL extraction, urine is first basified with 1% sodium hydroxide (NaOH) and then extracted with acetonitrile (ACN), yielding relatively low recoveries of only 17.7–20.9% (thus requiring internal standard correction for accurate quantification). For CYA extraction, urine is acidified with 1% FA followed by extraction with EtAc/IPA (v/v = 95/5), achieving recoveries of 70.1–70.9% [3]. Although this sequential method offers high selectivity for CYA, it is cumbersome to operate and results in unacceptably low recovery rates for MEL.

SPE is exclusively employed for complex urine samples due to its superior purification capability. In the analysis of bovine urine, Oasis MCX cartridges were used for MEL extraction and Oasis MAX cartridges for CYA extraction. This combination achieved recoveries of 74–96% and an RSD of <18%, effectively removing impurities derived from feed residues [11]. Nevertheless, the complexity and high cost of SPE limit its applicability in large-scale human urine analyses, where cost-efficiency and throughput are critical [8].

In conclusion, the selection of an appropriate extraction method must align with specific research requirements: two-step LLE is recommended for multi-component exposure assessment [27,37], sequential LLE should be chosen based on the need for single-component detection [3], and SPE can be used as an auxiliary technique for complex urine samples [8]. Future research directions may include optimizing solvent ratios or developing molecularly imprinted materials, with the goal of balancing extraction efficiency and detection sensitivity.

##### OPE Metabolites

Urine is a commonly used matrix for in vivo exposure monitoring of OPs, and its pretreatment requires addressing the dissociation of glucuronide-conjugated metabolites and correction for urine dilution effects (Table S2).

Hernandez-Castro et al. employed a combined SPE–enzymatic digestion technique for analyzing OPE metabolites in maternal urine. A 0.5 mL urine sample was spiked with an internal standard and 10 mM ammonium acetate buffer (pH = 5), then enriched using a STRATA-X-AW SPE column (60 mg, 3 cc). The column was sequentially eluted with 5% ammonia-methanol, methanol, and water to activate the column. After sample loading, the column was vacuum-dried for 3 min, followed by elution with 5% ammonia-methanol. The eluate was evaporated to dryness under nitrogen and redissolved in ACN. This method optimized extraction efficiency for polar metabolites in urine, achieving matrix-spiked recovery rates of 70.4–133% [51]. Kang et al. employed SPE–enzymatic hydrolysis for NHANES urine samples. After β-glucuronidase hydrolysis of 400 μL urine, purification occurred via STRATA-X-AW SPE column elution with 2% ammonia-methanol. This method achieved LODs as low as 0.05–0.16 ng/mL for chlorinated metabolites like BDCIPP and BCEP. Moreover, it employed a “covariate-corrected normalization method” to mitigate interference from traditional creatinine-based corrections in renal function-related studies [12].

### 3.2. Instrumental Analysis

#### 3.2.1. MEL and Its Derivatives

Currently, LC-MS/MS is the mainstream technique for the detection of MEL and its derivatives (Table S1). This approach combines separation using hydrophilic interaction liquid chromatography (HILIC) columns or C18 columns with electrospray ionization (ESI) in multiple reaction monitoring (MRM) mode, enabling high-sensitivity and high-specificity analysis.

The selection of chromatographic columns is guided by the polarity of target compounds and the properties of the sample matrix. Highly polar compounds such as MEL (Log *K*_ow_ = −1.37) and CYA (Log *K*_ow_ = −0.67) show weak retention on reversed-phase C18 columns, while HILIC columns enable excellent retention of highly polar compounds through liquid–liquid partitioning mediated by a water film on the surface of stationary phases (e.g., amide groups, silanol groups), significantly extending their retention times and thus becoming the primary choice for such analyses [7,8,47]. Zhu et al. employed a Luna HILIC column (100 mm × 3.0 mm, 3.0 μm) with a mobile phase of ACN and 5 mM ammonium formate (AmFm, pH 4.0, v/v = 9/1), achieving baseline separation of MEL, CYA, ammeline (AMN), and ammeline (AMD) with retention times of 5.0, 1.9, 6.0, and 3.4 min, respectively, and effectively avoiding interference from water-soluble impurities in urine [37]. Liu et al. used an ACQUITY UPLC^®^ BEH HILIC column (Waters, Milford, MA, USA) for the simultaneous separation of the four target analytes, which was specifically adapted to the analysis of high-water-content food simulants (water and 4% acetic acid) in tableware migration tests [47]. To resolve the co-elution of MEL and its metabolites in 24 h urine samples, Liu et al. selected a Waters ACQUITY UPLC BEH Amide column (1.7 μm, 2.1 × 100 mm, USA) with a mobile phase of 5 mM AmFm (pH 4.0) and ACN, providing a reliable separation basis for time-trend analysis of MEL exposure [27]. Due to the significant polarity difference between MEL and CYA, Zhang et al. designed a method with differentiated instrumental conditions: an Allure PFP Propyl column for MEL separation and an XBridge Phenyl column for CYA separation, ensuring effective separation of the two compounds with a total runtime of only 3 min (MEL: 3.5 min; CYA: 3 min) [3]. However, this method requires two different chromatographic columns, making it unsuitable for high-throughput analysis of complex samples. In the analysis of bovine urine, Zhang et al. also used a Luna HILIC column (100 mm × 3.0 mm, 3.0 μm particle size; Phenomenex, Torrance, CA, USA) and added a Betasil C18 guard column (20 mm × 2.1 mm, 5 μm particle size; Thermo Scientific, Waltham, MA, USA) in front of the analytical column to enhance MEL retention in more complex organic matrices (e.g., high-fat/high-organic-matter matrices), and acidic mobile phases were used to strengthen the interaction of CYA with the stationary phase [8].

The differences in chemical properties between MEL and its derivatives determine the selection of ionization modes, and currently, for the determination of total MEL (∑MEL), either switching between positive and negative ESI modes or separate detection in individual modes is used. ESI positive mode (ESI+) is suitable for the ionization of alkaline compounds such as MEL and AMN: Zhu et al. used ESI+ mode (data acquisition: 4.2–18 min) to detect MEL (*m*/*z* 127→85, 68) and AMN (*m*/*z* 128→86), with collision energies optimized to 20 V and 25 V, respectively, to maximize the intensity of product ions [37]. Zhang et al. set the ion spray voltage to 5500 V and the source temperature to 450 °C, with MRM transitions of *m*/*z* 127/68 (for quantification) and 127/85 (for qualification) for MEL, while the internal standard (^13^C_3_-^15^N_3_-MEL) was monitored at *m*/*z* 133/72, effectively correcting for ionization suppression caused by the urine matrix [8]. ESI negative mode (ESI-) is tailored for the ionization of acidic compounds such as CYA and AMD: Zhu et al. utilized ESI- mode (data acquisition: 0–4.2 min) for the detection of CYA (*m*/*z* 129→42) and AMD (*m*/*z* 127→85), and a reduced spray voltage (−4500 V) was used to minimize negative ion interference from organic acids (e.g., uric acid) in urine [37]. Zhang et al. optimized ESI- parameters for CYA as follows: ion spray voltage of −4500 V, source temperature of 500 °C, MRM transitions of *m*/*z* 128/42 (quantification) and 128/85 (qualification), and monitoring of the internal standard (^13^C_3_-^15^N_3_-CYA) at *m*/*z* 134/44 [3]. By switching ionization modes within different retention time windows, simultaneous detection of the four target analytes can be achieved with a single injection, improving efficiency by 50% compared to separate detection [27,37,47]. In contrast, Zhang et al.’s method did not use HILIC columns, requiring separate sample treatment for MEL and CYA and detection in different ionization modes; although this approach maintained high specificity, it resulted in a more cumbersome workflow [3].

#### 3.2.2. OPE Metabolites

Detection technologies for OPEs and their metabolites are primarily dominated by HPLC-MS/MS and UPLC-MS/MS, while Gas Chromatography–Mass Spectrometry (GC-MS/MS) is employed for the detection of volatile OPEs (Table S2).

HPLC-MS/MS has become the mainstream technique for bio-matrix analysis due to its suitability for highly polar, thermally unstable OPE metabolites. Hernandez-Castro et al. employed HPLC/quadrupole–quadrupole mass spectrometry (QqQ-MS) to detect nine metabolites in urine [51]. The chromatographic system comprised a Kinetex HILIC column (100 mm × 2.1 mm, 2.6 μm) coupled with a Betasil C18 guard column. The mobile phase consisted of a gradient elution of ACN-5 mM NH_4_Ac, with ESI and MRM mode for quantification. This method achieved a LOD of 0.0174 ng/mL for BDCIPP and demonstrated <10% quantitative deviation after matrix effect correction via isotope dilution [51]. Similarly, Kang et al. employed Agilent 1290 HPLC coupled with AB SCIEX QTRAP 5500 mass spectrometry, utilizing a C18 column (50 mm × 2.1 mm, 1.8 μm) for separation, ESI-ionization, and MRM mode detection. This approach yielded a LOD as low as 0.08 ng/mL (for BCEP) and eliminated urine matrix interference through matrix-matched standard curves [12]. In a longitudinal study of children’s urine, Yang et al. employed UPLC-MS/MS technology using a Waters BEH C18 column (50 mm × 2.1 mm, 1.7 μm) with a mobile phase gradient elution of methanol–water. The total run time was only 8 min, representing a 50% reduction compared to traditional HPLC. Highly selective detection was achieved through specific MRM channels [50]. For hydroxylated metabolites, Wang et al. employed UPLC–triple quadrupole mass spectrometry with ESI and optimized column extraction to achieve baseline separation from the parent metabolite DPHP, with a LOD of 0.04 ng/mL [52].

GC-MS/MS is suitable for volatile, thermally stable OPEs and is primarily used for environmental matrix detection. Zhang et al. employed GC-MS/MS to detect TCEP in dust using a DB-5MS column (30 m × 0.25 mm, 0.25 μm). The temperature program involved a 2 min hold at 40 °C followed by a 10 °C/min ramp to 300 °C. Ionization was performed in EI mode, with MRM mode used for quantification. This method achieved a LOD of 0.01 ng/g for TCEP. Matrix-spiked recovery experiments validated recovery rates ranging from 85% to 105% [26].

The ionization patterns of OPE metabolites depend on their structural characteristics. ESI^−^ is suitable for metabolites containing highly electronegative groups, such as chlorinated OPEs and phosphodiesters, which readily form [M-H]^−^ or [M + CH_3_COO]^−^ ions. For example, BDCIPP exhibits a parent ion at *m*/*z* 317 in ESI^−^ mode, with daughter ions selected at *m*/*z* 35 and *m*/*z* 175, corresponding to collision energies of 5 V and 17 V, respectively [51]. The parent ion of DPHP has a mass-to-charge ratio of 249, with daughter ions at *m*/*z* 155 and *m*/*z* 93. The collision energy (CE) settings are 15 V and 30 V [50]. ESI^+^ is suitable for metabolites containing amino or weakly polar groups, forming [M + H]^+^ ions. For example, the parent ion of TCEP is *m*/*z* 285, with daughter ions at *m*/*z* 99 and *m*/*z* 63, and CE voltages of 21 V and 29 V [12]. The core of MRM parameter optimization lies in balancing sensitivity and specificity by adjusting the CE and dwell time. For instance, when detecting low concentrations of BCIPHIPP (detection limit 0.01 ng/mL), Hammel et al. reduced the CE from 10 V to 4 V to minimize fragmentation of daughter ions, while simultaneously extending the dwell time to 100 ms, thereby improving the signal-to-noise ratio to over 30:1 [53]. Hernandez-Castro et al. increased the CE voltage to 30 V for high concentrations of DPHP and enhanced the ion intensity to avoid matrix interference [51]. Additionally, the selection of isotopic internal standards must align with the ionization mode of the target analyte. For instance, d_10_-BDCIPP was employed as the internal standard for BDCIPP, correcting matrix effects in ESI^−^ mode to stabilize recovery rates at 70–133% [51].

### 3.3. Method Validation

#### 3.3.1. Recovery

##### (a) MEL and Its Derivatives

The detection of MEL and its derivatives (CYA, AMN, and AMD) is significantly influenced by matrix type, pretreatment methods, and the polarity and acid–base properties of the compounds (Table S1). LLE is the primary method for urine extraction, with pH adjustment and internal standard calibration enhancing recovery stability. Based on the distinct polarity profiles of the four compounds, Zhu et al. employed a two-step LLE approach (alkalization for MEL and AMN detection, acidification for CYA and AMD detection). Following separation via HILIC, recovery rates for MEL, CYA, AMN, and AMD ranged from 69% to 94% at spiked levels of 1.0 μg/mL, 10 μg/mL, and 100 μg/mL. With RSD <12% [37], this method effectively counteracts the suppression of ionization efficiency by creatinine and organic acids in urine through gradient elution and isotope internal standardization (^13^C_3_-^15^N_3_-MEL/CYA), stabilizing recovery rates at 69–94%. This represents a significant improvement over traditional methods without internal standards. Zhang et al. treated MEL and CYA separately. After alkalization with 1% NaOH, the recovery rate of MEL extracted with ACN was only 17.7–20.9%. In contrast, after acidification with 1% FA, the recovery rate of CYA extracted with ACN/IPA (v/v, 95/5) reached 66.7–71.5% [3]. Despite the low recovery rate, the authors reported quantitative deviation within ±10% through ^13^C_3_-^15^N_3_-MEL internal standard calibration, meeting FDA bioanalytical validation requirements.

Liu et al. employed HILIC-LC-MS/MS in a 24 h dynamic monitoring of human urine, achieving MEL and CYA recovery rates of 72–88% across a broad concentration range of 1.2–230 ng/mL, with RSD < 10% [27], This study further confirms that even when urinary matrix components fluctuate due to dietary intake (e.g., postprandial increases in organic acids), long-term stability of recovery rates can be maintained by fixing extraction pH (alkalizing MEL to pH = 10 and acidifying CYA to pH = 3) and applying internal standard calibration. Zhu et al. detected recovery rates in bovine urine that were highly similar to those in human urine, with MEL and CYA recovery rates ranging from 74% to 96% and RSD < 18% [8]. This indicates that the impact of major interferents in urine matrices (such as proteins and electrolytes) on the extraction efficiency of MEL and its derivatives exhibits species universality, and a unified pretreatment method (combining LLE and HILIC) can be applied across species. LLE is suitable for simple polar matrices such as urine [54]. However, pH adjustment is required to enhance distribution efficiency. The complex composition and numerous interference sources in urine matrices can be mitigated through isotope internal standardization or SPE purification to offset their negative impact on recovery rates [3,6,8,37].

##### (b) OPE Metabolites

The recovery rate of OPE metabolites in urine is affected by multiple factors, including extraction materials, enzymatic digestion conditions, matrix effects, internal standard selection, and urine matrix characteristics (Table S2).

First, the type of SPE column significantly impacts the retention and elution efficiency of target compounds. Zhang et al. employed CNW P-WAX SPE columns for extracting eight OPE metabolites, achieving matrix-spiked recovery rates ranging from 78 ± 11% (BDCIPP) to 112 ± 16% (BBOEP) [55]. Although Hoffman et al. and Hammel et al. achieved effective enrichment of chlorinated and non-chlorinated metabolites using a StrataX-AW SPE column, the recovery of the internal standard d_12_-TCEP was inhibited by the urine matrix (34 ± 1.0%), which was significantly lower than that of the aqueous blank (55–73%), suggesting that the suitability of different SPE columns for metabolites of OPEs with large differences in polarity. This suggests that different SPE columns have different adaptability to metabolites of OPEs with different polarity (e.g., BCEP and DPHP), and that complex urine matrices are prone to reduce the recoveries through competitive adsorption or ionic inhibition [48,49].

Secondly, enzymatic conditions are the key to the hydrolysis of bound metabolites, and most of the existing studies have used the mixed system of β-glucuronidase and sulfate esterase, which was incubated at 37 °C overnight. For example, Yang et al. demonstrated that the recovery rate was 98–108% under this condition. Insufficient enzyme activity or insufficient incubation time will result in incomplete release of bound metabolites, which will significantly reduce the recovery rate [50]. In contrast, Hammel et al. found by analyzing a standard reference material (SRM 3673) that differences in enzymatic efficiency may lead to deviations in the recovery of hydroxylated metabolites such as BCIPHIPP from historical laboratory data [49].

Internal standard selection needs to match the metabolic behavior of the target. Hammel et al. used ^13^C_2_-DPhP as an internal standard to assess the recovery of aryl metabolites (e.g., DPhP), avoiding errors due to differences in extraction efficiency [49], while Zhang et al. pointed out that trace DBP (0.14 ± 0.08 ng/mL) in the blank samples should be deducted; otherwise the actual recovery would be overestimated [26].

#### 3.3.2. Limit of Detection (LOD) and Limit of Quantitation (LOQ)

##### (a) MEL and Its Derivatives

LOD and LOQ are the core indicators to evaluate the sensitivity of MEL and its derivatives (CYA, AMN, and AMD), which are synergistically regulated by the chromatographic separation, ionization parameters, pretreatment, matrix effects, and physicochemical properties of compounds (Table S1).

The degree of purification and concentration achieved during pretreatment directly determines the level of matrix interference and the enrichment efficiency of target analytes, serving as a core factor influencing LOD/LOQ. Zhang et al. reported LOQ values of 10 ng/mL for both MEL and CYA. The relatively high values stem primarily from the use of a single LLE step in pretreatment: MEL was treated with NaOH for alkalization, followed by EtAc addition, while CYA underwent acidification with FA before EtAc/IPA addition. The absence of SPE purification resulted in significant matrix interference, limiting detection sensitivity [3]. Zhu et al. used blank human urine to prepare matrix-matched standard curves to correct for the inhibition of ionization by creatinine (mean value of 160 mg/dL), and paired with isotopic internal standards to compensate for extraction losses, resulting in a LOQMEL control of 0.08 ng/mL [37]. For bovine urine with a complex matrix (mean creatinine value of 63 mg/dL), Zhu et al. removed impurities by intensive Oasis MAX/MCX SEP purification, and corrected for extraction losses by isotopic internal standards ^13^C_3_-MEL and ^15^N_3_-CYA, with LOQ_MEL_ and LOQ_CYA_ of 0.20 ng/mL and 0.12 ng/ mL [8]. Liu et al. significantly enhanced the target concentration by SPE purification and nitrogen blowing concentration, resulting in a low LOD_MEL_ of 0.03 ng/mL [27]. Liu et al. further optimized the pretreatment using distributed pH-adjusted extraction (5% NH_4_OH alkalinization and 1% FA acidification), Oasis MAX/MCX SPE purification, and 10-fold nitrogen-blowing concentration to achieve efficient enrichment of the target and deep removal of the substrate, resulting in a reduction of LOQ_MEL_ to 0.003 ng/mL [47].

The columns used by Zhang et al. (Allure PFP Propyl and XBridge Phenyl) have a particle size of 5 μm, which may result in an insufficient separation efficiency of the target, and therefore may lead to a high LOQ [3]. Zhu et al. used a 3 μm Luna HILIC column with a hydrophilic interaction mode suitable for the separation of polar targets, paired with a 5 mM AmFm buffer (pH 4.0) to optimize peak row symmetry and separation and to enhance LOQ [8,37]. Liu et al. and Liu et al. employed a 1.7 μm particle size UPLC BEH Amide/HILIC column combined with a 5 mM AmFm mobile phase to enhance the retention and ionization of polar substances, resulting in LODMEL and LOQMEL as low as 0.03 ng/mL [27] and 0.003 ng/mL [47].

The response value and background noise level of the target were optimized by mass spectrometry parameter optimization and ionization mode matching. Zhu et al. adjusted the CV of MEL to 30 eV and CYA to −30 eV to enhance the abundance of the characteristic ions MEL: *m*/*z* 127→85, CYA: *m*/*z* 128→42, and to reduce the LOQ [37]. Zhang et al. used mode switching (ESI+ mode MEL: 5500 V, ESI- mode CYA: −4500 V) to address the polarity difference between MEL and CYA, but the purification step was not optimized, and the sensitivity was still limited [3]. Zhu et al. continued the mode-switching strategy, using ESI- to detect CYA and AMD from 0–4.2 min, and ESI+ to detect MEL/AMN from 4.2–18 min, to reduce ion suppression [8,37]. Liu et al. used the MRM multistage fragmentation model to reduce the background noise interference, resulting in a lower LODMEL [27]. Liu et al. reported better hardware performance with SCIEX Triple Quad 6500, and mass spectrometry achieved LOQ_MEL_ as low as 0.003 ng/mL [47].

##### (b) OPE Metabolites

The LOD/LOQ of OPE metabolites in urine is related to sample pretreatment, instrumental technique, and matrix calibration (Table S2).

The enrichment and purification efficiency of the sample pretreatment process is the basis of the LOD/LOQ. Zhang et al. used a CNW P-WAX SPE column to optimize the elution system (3 × 400 μL 2% NH_4_OH-methanol (MeOH)) to achieve efficient enrichment of eight OPE metabolites, with the LOQ as low as 0.001–0.50 ng/mL, of which the LOQ of BDCIPP reached 0.001 ng/mL, and the LOQ of BDCIPP reached 0.001 ng/mL. The LOQ of BDCIPP was as low as 0.001 ng/mL, which was closely related to the high retention of chlorinated metabolites (e.g., BCEP) on the strong polarity SPE column [26]. Hoffman et al. used StrataX-AW solid-phase extraction columns, and the detection limits of BCIPP ranged from 0.136 to 0.333 ng/mL. The polarity of the solid-phase extraction columns matched with the target substances directly affects the purification of the matrix, and a high degree of sample purification enhances the lower limit of LOD [48].

The instrumental method is the core of determining the LOD/LOQ. Yang et al. used isotope dilution HPLC-MS/MS, Shimadzu HPLC-Q-Trap 5500, and optimized the ESI parameters to achieve the LOD as low as 0.1 ng/mL and *r*^2^ > 0.99 for BCEP and BDCIPP. The high selectivity of the MRM mode of triple quadrupole mass spectrometry for target ions is the key [50]. Kang et al. used AB Sciex 5500 Qtrap HPLC-ESI-MS/MS based on NHANES data, and the LOD (0.05–0.16 ng/mL) was slightly higher than that reported by Yang et al. [12], suggesting that target polarity and ionization efficiency further affect the instrument’s lower limit of detection.

Matrix effect correction also significantly affected LOD/LOQ accuracy. Hammel et al. counteracted matrix inhibition of ionization by adding stable isotope internal standards, such as ^13^C_2_-DPHP, to stabilize LOD in BCIPHIPP at 0.05 ng/mL, with a detection bias of <10% in the standard reference material (SRM 3673) [49]. In contrast, Hernandez-Castro et al. deducted the correction from the blank only, and although the LOD of BBOEP (0.0199 ng/mL) was lower, the CV of the actual samples detected at low concentrations was as high as 19%, which indicated that the insufficient correction reduced the reliability of the detection at low concentrations [51]. Hammel et al. increased the sample volume to 5 mL to enhance the enrichment of low-concentration urine from infants and reduced the LOQ of BCIPP to 0.07 ng/mL [49]. Zhang et al. showed that a sample volume of 2 mL was sufficient for LOQ <0.5 ng/mL in normal adult urine [26].

## 4. Exposure Characteristics and Health Risks of Multiple Classes of Contaminants in Urine

### 4.1. MEL and Its Derivatives

#### 4.1.1. Exposed Levels

**Figure 1:**
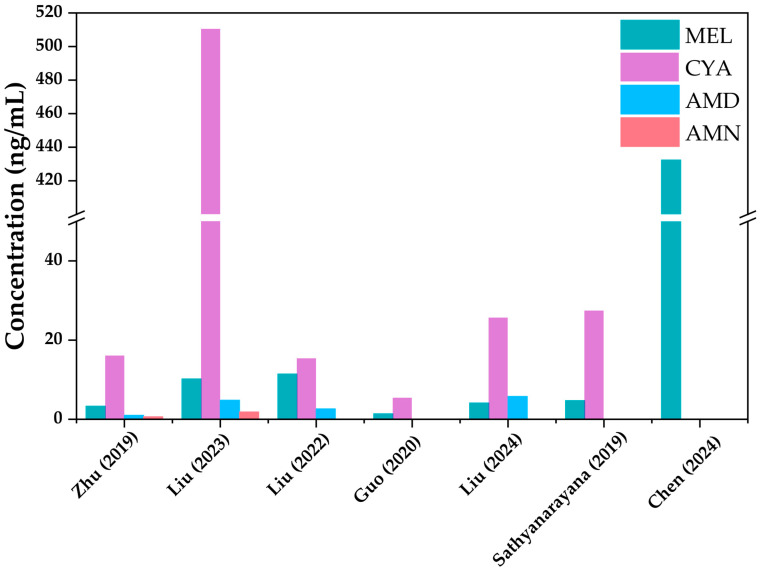
Concentration levels of MEL and its derivatives in published studies [27,37,47,57,58,59,62].

MELs are widely exposed in global populations, with the detection rates and concentrations varying significantly across different compounds and populations. Typically, CYA is the dominant component in urine, MEL is the second largest component, while AMD and AMN are detected at lower rates and percentages [37]. AMN was detected at significantly lower rates than other compounds in many studies, and exposure levels were not high enough to pose a health risk [47,56,57] (Table 2). Due to the rapid metabolism of MELs in humans, the focus needs to be on the effects of short-term acute exposure to MELs and CYA. The results of the concentrations of MEL and its derivatives in each study are shown in Figure 1.

Using NHANES 2003–2004 data to assess the renal effects of urinary MEL and CYA exposure in US adults, Guo et al. showed that the detection rates of MEL and CYA were 77.8% and 99.3%, respectively, suggesting that low-dose exposures were widespread [58]. The reported geometric concentration of CYA (GM_CYA_ = 5.34 ng/mL) was higher than that of MEL (GM_MEL_ = 1.40 ng/mL) [58]. In the U.S. GAPPS cohort, 100% of children aged 4–6 years were detected with MEL, 99.2% with AMD, and 87.8% with CYA, with mean concentrations of 6.1, 1.9, and 60.6 ng/mL, respectively [56]. In the Shanghai Suburban Adult Cohort and Biobank (SSACB) population in China, the detection rates of MEL, AMD, and CYA were 96.65%, 97.91%, and 97.07%, respectively [57]. MEL (32.74%) and CYA (57.93%) were the main components of ∑MEL [57]. The median concentration of CYA in the urine of Chinese students was 45.9 ng/mL, with CYA accounting for 74–80% of the ∑MEL concentration [37,47]. The concentration of MEL in the urine of Chinese melamine factory workers (432 ± 451 ng/mL) was significantly higher than that of the general control group (7.80 ± 11.3 ng/mL) [59].

**Table 2:** Summary of urinary concentrations, estimated daily intakes (EDI), and tolerable daily intakes (TDI) of MEL and its derivatives.

Chemicals	Concentration (ng/mL)	EDI (ng/kg bw/day)	TDI (μg/kg bw/day)	Ref.
$\sum_{4}MEL$ MEL, CYA, AMN, AMD	Mean: MEL: 3.3; CYA: 16; AMD: 0.99; AMN: 0.62; $\sum_{4}MEL$: 20	Mean: MEL: 65.5 CYA: 315	3.15 [60] 8.1 [61] 63 (US FDA)	[37]
$\sum_{4}MEL$ MEL, CYA, AMN, AMD	Mean: MEL: 10.19 ± 12.57 AMN: 1.83 ± 0.98 AMD: 4.80 ± 2.97 CYA: 510.53 ± 621.75	13.84–5973.49	3.150 [60] 8.1 [61] 63 (US FDA)	[27]
$\sum_{4}MEL$ MEL, CYA, AMN, AMD	Median: MEL: 11.41 AMN: nd AMD: 2.64 CYA: 15.30 ∑MEL: 35.02	Median: MEL: 0.26 AMN: nd AMD: 0.06 CYA: 0.32 ∑MEL: 0.76	3.15 [60] 8.1 [61] 63 (US FDA)	[57]
$\sum_{2}MEL$ MEL, CYA	GM: MEL: 1.40 CYA: 5.34	Median: MEL: 60	3.15 [60] 8.1 [61] 63 (US FDA)	[58]
$\sum_{4}MEL$ MEL, CYA, AMN, AMD	Median: MEL: 4.10; CYA: 25.6; AMD: 5.78; AMN: nd	Median: MEL: 327 CYA: 656	MEL: 200 (WHO) CYA: 150 (WHO)	[47]
$\sum_{2}MEL$ MEL, CYA	Median: MEL: 4.7; CYA: 27.4	MEL: 12–250 (children) CYA: 20–500 (children)	MEL: 3.15 CYA: 2.50	[62]
MEL	Mean: Stone patients: 12.25 ± 25.42 MEL factory workers: 432.35 ± 451.26 Steel workers: 7.80 ± 11.28	Median: Stone patients: 450 MEL factory workers: 23,360 Steel workers: 350	200 (WHO) 63 (FDA)	[59]

Abbreviation: nd, not detected or <LOQ.

#### 4.1.2. Health Risks of MEL and Its Derivatives 

The estimated daily intake of MELs varies significantly among populations, with some populations approaching or exceeding safety thresholds. Diet was the main route of MEL intake, with dairy (3.81–42.6 ng/kg bw/day), cereals (3.14–10.20 ng/kg bw/day), and meat (2.12–7.03 ng/kg bw/day) contributing the most to exposure [6]. Postprandial monitoring of Chinese adults showed that MEL concentrations peaked at 3 h postprandial (mean 8.6 ng/mL, 22 times higher than preprandial) and that the CDI for MEL ranged from 13.8 to 5970 ng/kg bw/day (mean 557 ng/kg bw/day) based on a strict tolerable daily intake (TDI) of 3.15 μg/kg bw/day [27,60]. About 3.8% of adults in suburban Shanghai, China, had estimated daily intakes of CYA (EDI_CYA_) above threshold (TDI_CYA_ = 2.50 μg/kg bw/day) [57]. Higher EDIs for MEL were found in special populations (1100 ng/kg bw/day for kidney stone patients and 43,960 ng/kg bw/day for melamine plant workers) [59]. The EDI_CYA_ of children in the United States was 656 ng/kg bw/day [62]. EDI_MEL_ and EDI_CYA_ of college students in China were 1.9–3.3 times higher than those of the general population, respectively [47].

MELs exposure differs significantly by gender, age, and occupational/lifestyle factors. Urinary ∑MEL concentrations were significantly higher in females (4.3 ng/mL) and older age groups (4.0 ng/mL) than in males (2.5 ng/mL) and younger age groups (2.1 ng/mL) [37]. The inter-day variability of compounds was moderately predictable after correction for creatinine (intragroup correlation coefficients ICCs = 0.541–0.763) [37]. Occupation and lifestyle further exacerbate the disparity; for example, melamine plant workers have much higher MEL exposures than the general population due to occupational exposures [59]. Significantly higher exposure levels in college students due to high-frequency use of melamine-containing tableware [47]. Children, as a sensitive population, are not exposed to high single doses, but long-term low-dose exposures present unique risks [56,62]. Infants, toddlers, and children are sensitive populations to MEL and its derivatives. Indoor dust and nap mats serve as exposure sources, with median MEL concentrations of 3210 ng/g and 27.2 ng/g, respectively [63]. Daily intake via dust exposure (EDI) was calculated as EDI_MEL_ = 3.40 ng/kg bw/day and EDI_CYA_ = 1.23 ng/kg bw/day [63].

Zhu et al. showed that diet is the main route of MEL intake, with higher exposure via dairy (3.81–42.6 ng/kg bw/day), grains (3.14–10.2 ng/kg bw/day), and meat (2.12–7.03 ng/kg bw/day) [6]. Postprandial MEL concentration peaks further confirm that dietary intake is the primary source of acute exposure [27]. Melamine tableware contributes 66.9% and 36.2% to CYA and AMD exposure among students, respectively, making it a significant source of daily exposure [47].

Health effect assessments based on the hazard quotient (HQ) and hazard index (HI) indicate significant variations in risk across different populations. For U.S. adults adhering to the stringent TDI (3.15 μg/kg bw/day) standard, HQ_MEL_ < 1, and MEL/CYA exposure shows no significant association with urinary albumin to creatinine ratio (UACR), renal impairment, or hypertension, indicating low short-term risk [60]. In the general population, the CDI_MEL_ and CDI_CYA_ were at least 10 times lower than the TDI, posing no acute risk but requiring vigilance for potential renal risks from long-term low-dose exposure [37]. However, the risk in high-risk populations is prominent: in China, 6.21% of students with HQ_MEL_ > 1 and 7.59% with HQ_CYA_ > 1 [47]. In suburban Shanghai, China, about 5.0% adults showed HI_∑MEL_ >1 on a strict TDI (3.150 μg/kg bw/day) basis [57]. A study showed that 2 adults had HQ_MEL_ >1 after a meal [27]. Some patients with kidney stones HQ_MEL_ = 1.25 [59]. Melamine plant workers were at risk of kidney damage with HQ_MEL_ = 1.93 [59]. MEL exposure in melamine plant workers was associated with elevated levels of urinary N-acetyl-β-D-aminoglucosidase (a marker of kidney injury) [59]. CYA exposure was associated with elevated kidney injury molecule 1 (KIM 1), a marker of early kidney injury, in U.S. children despite HQ_CYA_ < 1 [62].

These results have important public health implications. First, children in the United States, students in China, and workers in melamine factories are high-risk groups, and the use of melamine tableware by children should be restricted, and occupational protection in factories should be strengthened [47,56,59,62]. Second, regulatory measures should be strengthened to control melamine exposure, including restricting its use in food-contact materials, establishing safety standards for melamine-based tableware (e.g., prohibiting use at high temperatures), and reducing dietary and household exposures [6,27,47]. Third, in view of the exceedance of HQ/HI in some populations in China, it is necessary to optimize the TDI/Reference Dose (RfD) of MELs by combining it with the local exposure characteristics, to avoid the underestimation of the local risk by the general standard [57,58]. Given the association of low-dose MEL/CYA exposures with early markers of kidney injury, long-term cohorts (e.g., pediatric renal development cohort) need to be established to fill the “low-dose–long-term effects” research gap [59,62]. Finally, public education campaigns should be implemented to promote the safe use of melamine tableware (e.g., avoiding contact with hot food), encourage rational consumption of high-exposure foods (such as processed dairy products and grains), and enhance the population’s capacity for autonomous risk avoidance [6,47].

### 4.2. OPE Metabolites

#### 4.2.1. Exposed Levels

**Figure 2:**
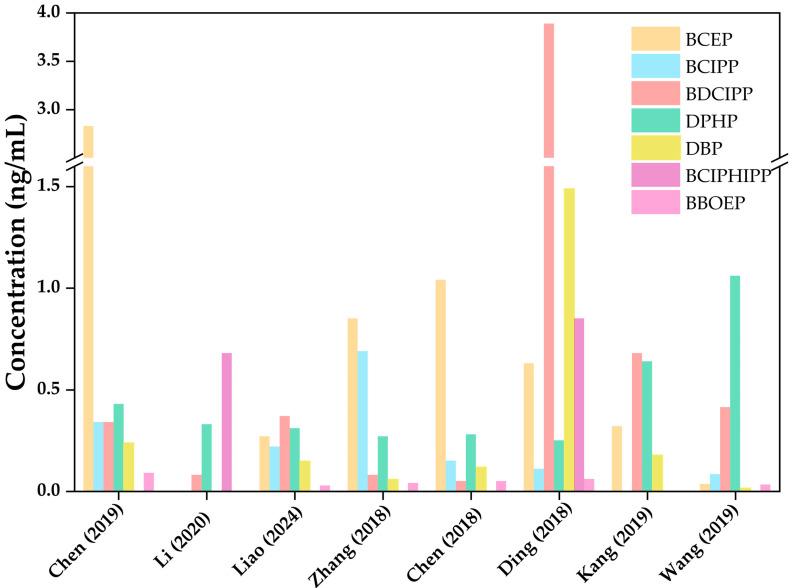
Concentration levels of OPE metabolites in published studies, listing only the seven OPE metabolites with high detection rates [12,53,64,68,69,71,72,73].

As widely used flame retardants and plasticizers, human exposure and health risk of OPEs have become a research hotspot in the field of environmental health, and urinary mOPEs are the key biomarkers for assessing human exposure to OPEs (Table 3). The concentrations of OPE metabolites in each study are shown in Figure 2. Existing studies have shown that global variations exist in the homologue profiles of urinary OPE metabolites: BDCIPP and DPhP are the most frequently detected metabolites across regions; DNBP and other related metabolites are commonly found in samples from Asia; while isopropyl diphenyl phosphate and tert-butylphenyl phenyl phosphate are more prevalent in North American samples, these differences in homologue profiles reflect regional disparities in regulations, production and consumption patterns, and lifestyles among different countries [4].

In Shenzhen, China, researchers examined the urine of 84 primiparous women and found that among the 8 mOPEs, BCEP was detected in 95% of the cases, with a median concentration of 1.32 μg/L, which was the most predominant mOPE [64]. The median concentration of ∑_8_mOPEs was 2.78 ng/mL, with BCEP accounting for 57.6% of the total [64], which was significantly higher than that of children in Germany (median concentration of BCEP 0.20 ng/mL) [65] and pregnant women in Canada (median concentration of BCEP 0.46 ng/mL) [66], as well as that of children in Australian (<0.02 mg/L) [67]. With the concentration of 1.32 ng/mL, BCEP accounted for 57.6% of the total metabolite burden [64]. This disparity may reflect elevated environmental exposure to TCEP, the parent compound of BCEP, which is prevalent in indoor dust in South China [64].

In a study of 46 residents from eight provinces in China, hydroxylated OPE metabolites (OH-OPEs) were the predominant urinary metabolites. 1-Hydroxy-2-propyl bis(1-chloro-2-propyl) phosphate (BCIPHIPP) was detected in 89.1% of samples (median: 0.68 ng/mL), and bis(2-butoxyethyl)-2-hydroxyethyl phosphate (BBOEHEP) in 54.3%. The total molar concentration of OH-OPEs (4.65 nM) exceeded that of conventional mOPEs (3.84 nM), indicating that hydroxylated metabolites play a significant role in assessing human exposure to OPEs [53].

A comparative study in Hong Kong, China, involving 101 e-waste workers and 100 office workers, found no significant difference in total ∑mOPE levels between the two groups (both ~1.86 μg/L). However, urinary diphenyl phosphate (DPHP) was significantly higher in e-waste workers (median: 0.31 ng/mL) than in office workers (0.25 ng/mL), whereas bis(1-chloro-2-isopropyl) 1-propyl phosphate (BCIPP) was higher in office workers (0.22 ng/mL). This suggests that occupational settings may influence exposure to specific OPE metabolites, despite similar overall burdens [68].

In a separate study of 19 residents in New York State, USA, DPHP (median: 919 pg/mL) and bis(1,3-dichloro-2-propyl) phosphate (BDCIPP, median: 359 pg/mL) were the predominant mOPEs. Females had significantly higher urinary concentrations of DPHP and BDCIPP than males, and individuals with obesity exhibited generally higher mOPE levels compared to those with normal BMI. These findings indicate that gender and physiological factors, such as body mass index, may modulate OPE exposure or internal distribution [69]. In California, the urinary levels of DPhP and BDCIPP in children are 5.9-fold and 15-fold higher than those in their mothers, respectively [70]. In urine samples from the general population participating in the National Health and Nutrition Examination Survey (NHANES) 2013–2014, the detection rates of DPhP, BDCIPP, bis(2-chloroethyl) phosphate (BCEP), and DNBP all exceed 75%, with geometric mean concentrations of 0.71, 0.69, 0.38, and 0.16 ng/mL, respectively [12].

**Table 3:** Urinary concentrations, estimated daily intakes (EDI), and tolerable daily intakes (TDI) of OPEs and their metabolites.

Chemicals	Concentration (ng/mL)	EDI (ng/kg bw/day)	TDI (μg/kg bw/day)	Ref.
OPEs metabolites				
$\sum_{8}OPEmetabolites$ BBOEP, BCEP, BCIPP, BDCIPP, DBP, DoCP, DpCP, DPHP	Mean: BBOEP: 0.09; BCEP: 2.83; BCIPP: 0.34 BDCIPP: 0.34; DBP: 0.24; DoCP: 0.09; DpCP: 0.09; DPHP: 0.43	Mean: TBOEP: 40 TCEP: 1020 TCIPP: 80 TDCIPP: 70 TNBP: 60 TCP: 20 TPHP: 110	TBOEP: 1.5 TCEP: 2.2 TCIPP: 5.0 TDCIPP: 1.5 TNBP: 2.4 TCP: 1.3 TPHP: 7.0 (the European Food Safety Authority)	[64]
$\sum_{11}OPEmetabolites$ DNBP, BCIPP, DPHP, BBOEP, BDCIPP, EHPHP, BCIPHIPP, 4-OH-DPHP, BBOEHEP, 5-OH-EHDPHP, TCEP	Median: DNBP: nd; BCIPP: nd; DPHP: 0.33 BBOEP: nd; BDCIPP: 0.08; EHPHP: 0.57; BCIPHIPP: 0.68; 4-OH-DPHP: nd BBOEHEP: nd; 5-OH-EHDPHP: 0.004 TCEP: nd	Median: EHDPHP: 273 TCIPP: 146 TDCIPP: 21.5 TPHP: 199 TBOEP: 0.52	TMP: 10 TNBP: 10 TCIPP: 10 TCEP: 7 TDCIPP: 20 TEHP: 100 TMPP: 20 DMMP: 60 (USEPA)	[53]
$\sum_{8}OPEmetabolites$ BCEP, BCIPP, BDCIPP, BBOEP, DBP, DoCP, DpCP, DPHP	Median: BCEP: 0.24–0.27; BCIPP: 0.16–0.22; BDCIPP: 0.33–0.37; BBOEP: 0.026–0.028; DBP: 0.13–0.15; DoCP: 0.058–0.068; DpCP: 0.043–0.050; DPHP: 0.25–0.31	Median: TBOEP: 6.07; TCEP: 53–57; TCIPP: 35–54; TDCIPP: 76–86; TNBP: 33–27; TCP: 27–23; TPHP: 68–51	TBOEP: 1.5 TCEP: 2.2 TCIPP: 8.0 TDCIPP: 1.5 TNBP: 2.4 TCP: 1.3 TPHP: 7.0	[68]
$\sum_{8}OPEmetabolites$ BCEP, BCIPP, BDCIPP, BBOEP, DBP, DOCP, DPCP, DPHP	Median: BCEP: 0.85; BCIPP: 0.69; BDCIPP: 0.08; BBOEP: 0.04; DBP: 0.06; DOCP + DPCP: 0.004; DPHP: 0.27	Median: TECP: 485; TCIPP: 80.5; TDCIPP: 8.11; TPHP: 119; TBOEP: 2.06	TCEP: 2.2 TCIPP: 8.0 TDCIPP: 1.5 TPHP: 7.0 TBOEP: 1.5	[71]
$\sum_{8}OPEmetabolites$ BBOEP, BCEP, BCIPP, BDCIPP, DBP, DOCP, DPCP, DPHP	Median: BCEP: 1.04; DPHP: 0.28; BCIPP: 0.15; DBP: 0.12; BBOEP: 0.05; BDCIPP: 0.05; DOCP + DPCP: 0.02	Median: TECP: 228; TPHP: 33.1; TCIPP: 22.6; TNBP: 19.2; TBOEP: 12.9; TDCIPP: 6.47; TCP: 2.78	TBOEP: 1.5 TCEP: 2.2 TCIPP: 8 TDCIPP: 1.5 TNBP: 2.4 TCP: 1.3 TPHP: 7	[72]
$\sum_{10}OPEmetabolites$ DBP, BCEP, BCIPP, DPHP, BBOEP, BDCIPP, BCIPHIPP, OH-DPHP, BBOEHEP, TCEP	Median: BDCIPP: 3.89; DBP: 1.49; BCIPHIPP: 0.85; TCEP: 0.17; DPHP: 0.25; BCIPP: 0.11; BBOEP: 0.06; OH-DPHP: nd; BCEP: 0.63; BBOEHEP: nd	Median: TDCIPP: 110; TNBP: 43; TCIPP: 25; TCEP: 21	TDCIPP: 1.5 TNBP: 2.4 TCIPP: 8.0 TCEP: 2.2 TPHP: 7.0	[73]
$\sum_{4}OPEmetabolites$ DPHP, BDCIPP, BCEP, DNBP	Median: DPHP: 0.64; BDCIPP: 0.68; BCEP: 0.32; DNBP: 0.18	-	-	[12]
$\sum_{11}OPEmetabolites$ DEP, DPRP, DNBP, DIBP, BBOEP, BEHP, BCEP, BCIPP, BDCIPP, DPHP, BMPP	DEP: 0.348; DPRP: 0.015; DNBP: 0.017; DIBP: 0.049; BBOEP: 0.033; BEHP: 0.013; BCEP: 0.035; BCIPP: 0.084; BDCIPP: 0.414; DPHP: 1.060; BMPP: 0.042	TEP: 24; TNBP: 1.09; TIBP: 2.49 TBOEP: 2.10; TEHP: 1.01; TCEP: 5.97; TCIPP: 0.34; TDCIPP: 8.51; TPHP: 19.4	TNBP: 80 TBOEP: 50 TCEP: 200 TDCIPP: 20	[69]

Abbreviation: nd, not detected or <LOQ.

#### 4.2.2. Health Risks

Multiple studies have evaluated the estimated daily intakes (EDIs) and health risks of OPE exposure across diverse populations in China and the United States. In a multi-provincial study of Chinese adults, OPE exposure EDIs ranged from 0.06 to 273 ng/kg bw/day, with bis(2-ethylhexyl) diphenyl phosphate (EHDPHP) contributing the highest median EDI (273 ng/kg bw/day). Despite this, hazard quotients (HQs) for all individual OPEs were below 0.22, indicating low immediate health risk [53].

In Hong Kong, the median electronic waste exposure index (EDI) for electronic waste processing workers was 104 ng/kg bw/day, while that for office workers was 89.9 ng/kg bw/day. Only 2% of electronic waste processing workers and 7% of office workers had a hazard index (HI) >1, indicating overall low risk. However, the study identified diphenyl phosphate (DPHP) as a key factor causing oxidative DNA damage—detected via 8-hydroxy-2′-deoxyguanosine (8-OHdG), a direct, specific biomarker for oxidative DNA stress damage. 8-OHdG possesses unique biochemical characteristics [74]. Furthermore, linear regression, quantile g calculations, and Bayesian kernel machine regression models all consistently confirmed an association between DPHP and elevated 8-OHdG levels. This further substantiates that 8-OHdG specifically reflects DPHP-induced oxidative DNA damage rather than non-specific oxidative stress [68].

For residents of New York State, the median EDI of ∑OPEs was 65.3 ng/kg bw/day, with significantly higher exposure in women (81.9 ng/kg bw/day) than in men (53.9 ng/kg bw/day). Nevertheless, HQs for all OPEs remained well below safety thresholds [69].

In adolescents from Hangzhou, China, BDCIPP was reported as the predominant OPE metabolite (median: 3.89 ng/mL), with a median estimated daily intake of TDCIPP (EDITDCIPP) at 110 ng/kg bw/day. All hazard quotients were below 1, indicating low acute health risk [73].

Among Chinese children, exposure levels varied by age group. In infants and toddlers (0–5 years), metabolite detection rates ranged from 60% to 100%, with high median urinary concentrations of BCEP (0.85 ng/mL), BCIPP (0.69 ng/mL), and DPHP (0.27 ng/mL). Parental EDIs for TCEP and TPHP reached 485 and 119 ng/kg bw/day, respectively, and 12% of children exceeded HI > 1, primarily due to TCEP, TCIPP, and TPHP [71].

In southern Chinese children aged 6–14 years, median urinary BCEP and DPHP concentrations were 1.04 and 0.28 ng/mL, corresponding to EDIs of 228 and 33.1 ng/kg bw/day for TCEP and TPHP, respectively. While 95% of individuals had HQ < 1, the high internal doses suggest potential concern for chronic exposure [72].

Collectively, these findings indicate widespread but generally low-risk OPE exposure across populations. However, subgroups—including young children and those with high TCEP/TPHP intake—show elevated EDIs and HI > 1 in a notable proportion, warranting attention for long-term health effects [64,71].

## 5. Discussion

This review systematically summarizes the current research progress in urine biomonitoring of MEL, its derivatives, and OPE metabolites, covering analytical methods, exposure characterization, and health risk assessment. The results not only reveal the widespread low-dose exposure to these pollutants in the general population but also highlight the key challenges and future research directions in this field. From the point of view of analytical methods, the pretreatment and instrumental detection of MEL and OPE metabolites in urine have formed a relatively mature technical system, but the applicability of the methods still differs significantly. For MELs, LLE combined with HILIC-LC-MS/MS has become the mainstream solution because of its stable structure and the lack of need for enzymatic treatment. This method can effectively solve the problem of poor retention of strong polar compounds such as CYA on reversed-phase columns, and the recoveries were maintained at a stable level of 69–96% with the lowest LOQ of 0.003 ng/mL. However, the two-step LLE process for MEL/AMN and CYA/AMD, which requires separate acid and base treatments, increases the operational complexity, and there is a strong need to develop high-throughput pre-treatment technologies such as automated SPEs to improve analytical efficiency. For OPE metabolites, the enzymatic step of β-glucuronidase/sulfate lyase combination is a key prerequisite to avoid underestimation of the exposure level, and the selection of SPE columns (e.g., Strata X-AW, CNW P-WAX) has a direct impact on the purification efficiency, and the LOQ of BDCIPP can be reduced to 0.001 ng/mL through optimization of the elution system. However, the interference of proteins, salts, and other components in the urine matrix on the ionization efficiency still needs to be strictly corrected by isotopic internal standards, which limits the comprehensiveness of the exposure assessment.

In terms of exposure characteristics, urinary concentrations of MEL and OPE metabolites showed significant spatial, population and temporal differences. Globally, the detection rates of MEL and CYA in the urine of the general population exceeded 97%, with CYA accounting for 66.2–80% of the total MEL (ΣMEL), which is closely related to its widespread use as a disinfectant and chlorine stabilizer [32,37]. The median concentration of ΣMEL in urine was slightly higher in the Chinese population (13–49 ng/mL) [27,47] than in the American population (20 ng/mL) [37], which may be related to the differences in dietary structure and the use of melamine tableware. Regional differences in OPE metabolites were more prominent: BCEP was the dominant component in the urine of primigravid women in Shenzhen (57.6% of ΣOPE metabolites) [64], whereas DPHP and BDCIPP predominated in the urine of residents of New York, USA [69]. Differences in the population were mainly in sensitive groups: children generally had higher concentrations of MEL and OPE metabolites in urine than adults, and the median concentration (0.31 ng/mL) of DPHP in the urine of e-waste workers in Hong Kong, China [68], was significantly higher than that of the office population (0.25 ng/mL) [68], suggesting the influence of occupational exposure. In the time dimension, the MEL concentration in urine peaked 3 h after a meal (8.6 ng/mL), which was 22.05 times the preprandial level, confirming that diet is a key source of exposure. Swimming behavior can lead to an abnormal short-term increase in urinary CYA concentration (25.07 times the pre-swimming level) [11], which suggests that biomonitoring needs to take into account the special exposure scenarios.

The health risk assessment showed that the current exposure levels of MEL and OPE are generally in the safe range, but the potential chronic risk should not be ignored. For MEL, based on the most stringent tolerable daily intake (TDI = 3.15 μg/kg bw/day), the majority of the general population had HQs < 0.2, but there were still 2–6.21% of individuals with HQs > 1, which were mainly found in the sensitive groups such as students using melamine tableware and the highly diet-exposed population [6,47]. Of concern: 12% of the primiparous HI >1 in Shenzhen [64], and DPhP was the main contributor to oxidative stress injury (elevated 8-OHdG) in e-waste practitioners [68]. It is important to note that existing risk assessments are mostly based on acute toxicity data, and there is insufficient epidemiological evidence for potential endocrine disrupting effects (e.g., disruption of thyroid hormone secretion) and neurotoxicity in chronic low-dose exposures, in particular, synergistic nephrotoxicity (formation of renal crystals) and co-toxicity between MEL and CYA and between metabolites of different OPEs need to be further clarified in a long-term cohort study.

## 6. Conclusions

Urine biomonitoring has become an effective tool for assessing MEL and OPE exposure in vivo, but three key issues still need to be resolved: first, standardizing analytical methods, such as unifying enzyme activity parameters and matrix effect correction strategies, to improve data comparability; second, expanding the range of target compounds, such as hydroxylated OPE metabolites and AMN/AMD, to achieve a comprehensive exposure assessment; and third, strengthening research on the correlation between exposure levels and early health effects to provide a more scientific basis for setting health protection standards. With the continuous improvement of testing technology and the accumulation of epidemiological data, it is expected that a more accurate and comprehensive human exposure–health risk evaluation system will be established for these emerging pollutants.

## Figures and Tables

**Table 1:** Names, abbreviations (Abb.), molecular formula (MF), CAS number (CAS No.), and structural formula of common MELs and their derivatives, OPEs and their metabolites.

Chemicals	Abb.	MF	CAS No.	Structural Formula
Melamine and Its Derivatives				
Melamine	MEL	C_3_H_6_N_6_	108-78-1	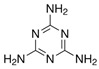
Cyanuric Acid	CYA	C_3_N_3_(OH)_3_	108-80-5	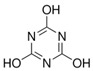
Ammeline	AMN	C_3_H_5_N_5_O	645-92-1	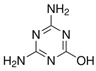
Ammelide	AMD	C_3_H_4_N_4_O_2_	645-93-2	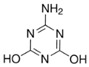
OPEs				
Triethyl phosphate	TEP	C_6_H_15_O_4_P	78-40-0	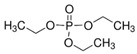
Tributyl phosphate	TNBP	C_12_H_27_O_4_P	126-73-8	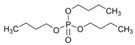
Tris(2-chloroethyl) phosphate	TCEP	C_6_H_12_Cl_3_O_4_P	115-96-8	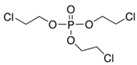
Tripropyl phosphate	TPP	C_9_H_21_O_4_P	513-08-6	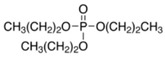
Triphenyl phosphate	TPHP	C_18_H_15_O_4_P	115-86-6	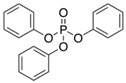
Tris(1,3-dichloro-2-propyl) phosphate	TDCIPP	C_9_H_15_C_l6_O_4_P	13674-87-8	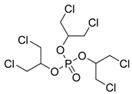
OPE metabolites				
di-n-butyl phosphate	DNBP	C_8_H_19_O_4_P	107-66-4	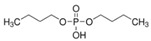
di-iso-butyl phosphate	DIBP	C_8_H_19_O_4_P	6303-30-6	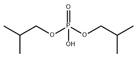
diphenyl phosphate	DPHP	C_12_H_11_O_4_P	838-85-7	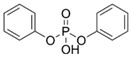
bis(1,3-dichloro-2-propyl) phosphate	BDCIPP	C_6_H_11_Cl_4_O_4_P	72236-72-7	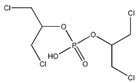

## Data Availability

The data will be made available upon request.

## Supplementary Materials

The following supporting information can be downloaded at: https://www.mdpi.com/article/10.3390/toxics13110950/s1, Table S1: Analytical methods for the determination of MEL and its derivatives in human urine. Table S2: Analytical methods for the determination of OPEs and their metabolites in human urine.
