# Supplementary material for: Internal Exposure Levels and Health Risk Assessment of Melamine and Organophosphate Metabolites in Urine: Research Progress and Prospects
Source: Toxics. 2025 Nov 4;13(11):950. doi: 10.3390/toxics13110950 (PMC12656575; doi:10.3390/toxics13110950)
Supplement: Supplementary file 1 [file toxics-13-00950-s001.zip › toxics-3907279-supplementary.pdf]

Supplementary Material: Internal Exposure Levels and Health Risk Assessment of Melamine and Organophosphate Metabolites in Urine: Research Progress and Prospects

Qu Zhang [1], Qi Jiang [1], Xin-Hong Wang [1], Liang Wang [2], Mei-Hua Tian [1], Da-Zhong Chen [1], Chun-Yan Huo [1, 2]*, Wen-long Li [1]*

[1] College of the Environment and Ecology, Xiamen University, Xiamen, 361102, China

[2] Third Institute of Oceanography, Ministry of Natural Resources, Xiamen 361005, China

*Corresponding authors. Chun-Yan Huo, e-mail address: huochunyan1119@163.com. Wenlong Li, e-mail address: wenlong.li@xmu.edu.cn.

Content

Supplementary Material: Internal Exposure Levels and Health Risk Assessment of Melamine and Organophosphate Metabolites in Urine: Research Progress and Prospects I
Content.......... 2
S1. Supplementary Tables.......... 3
Table S1. Analytical methods for the determination of MEL and its derivatives in human urine. 3
Table S2. Analytical methods for the determination of OPEs and their metabolites in human urine. 6
Reference 8

S1. Supplementary Tables

Table S1. Analytical methods for the determination of MEL and its derivatives in human urine.

Analyte	Urine volume	Enzyme Type	Sample preparation	Sample extraction	Instrument method	Recovery rate (%)	LOD/LOQ (ng/mL)	Ref.
MEL and its derivatives								
$\sum_4 MEL$<br>MEL, CYA, AMN, AMD	Two 250 µL aliquots per sample	None	Acidified with 50 µL of 1 % (v/v) FA and alkalized with 5 % (v/v) NH4OH, respectively;<br>5.0 ng of the IS mixture was added separately.	LLE<br>Acidified/alkalized samples were extracted twice with 2 mL EtAc/IPA (v/v, 95/5);<br>ACN/5 mM AmFm (pH 4.0, v/v, 9:1) was re-solubilized.	HPLC-MS/MS<br>LC column: Luna HILIC column (100 mm × 3.0 mm, 3.0 µm)<br>Guard column: Betasil C18 guard column (20 mm × 2.1 mm, 5 µm)<br>Mobile phase: ACN and 5 mM AmFm (pH 4.0)<br>Injection Volume: 2.0 µL<br>Ionization mode: Negative (CYA/AMD); Positive (MEL/AMN)	69-94 %	LOQ:<br>MEL: 0.08<br>AMN: 0.15<br>AMD: 0.10<br>CYA: 0.20	[1]
$\sum_2 MEL$<br>MEL, CYA	Two 100 µL aliquots per sample	None	After adding 10 µL of IS mixture to both samples, 20 µL of 1 % (v/v) FA acidification or 1 % (v/v) NaOH alkalinization was added.	LLE<br>One sample was extracted with 1 mL of EtAc; the organic phase was extracted with 100 µL of 0.1% (v/v) FA, and the aqueous phase was used for the detection of MEL.	HPLC-MS/MS<br>MEL:<br>LC column: Allure PFP Propyl (100 mm×2.1 mm I.D., 5 µm) + C_{18} guard column (4.0 mm×3.0 mm I.D., 5 µm)<br>Mobile phase: 50 % ACN/50 % MeOH (0.02 % FA) and 0.02 % FA solution<br>CYA:	MEL: 17.7-20.9 %<br>CYA: 66.7-71.5 %	LOQ: 10	[2]

				The other sample was extracted with 1 mL of EtAc/IPA (v/v, 95/5), and the aqueous phase was re-solubilized with 100 μL of 70 % MeOH for the detection of CYA.	LC column: XBridge Phenyl (150 mm×2.1 mm I.D., 5 μm) + C_{18} guard column<br>Mobile phase: MeOH (0.01 % FA)<br>Injection volume: 5 μL<br>Ionization mode: Negative (CYA); Positive (MEL)			
$\sum_4 MEL$<br>MEL, CYA, AMN, AMD	300 μL	None	After adding 5 ng of the IS mixture, it was alkalinized or acidified with 60 μL of 5 % (v/v) NH_4OH and 1 % (v/v) FA, respectively.	LLE<br>Alkalization/acidification followed by 2 extractions each with 2 mL EtAc/IPA (v/v, 95/5);<br>250 μL ACN/5 mM AmFm (pH 4.0, v/v, 9/1) re-solubilized.	UPLC-MS/MS<br>LC column: ACQUITY UPLC®<br>BEH HILIC column (Waters, Milford, MA, USA)<br>Mobile phase: water and ACN/5 mM AmFm (pH 4.0)<br>Injection volume: 5 μL<br>Ionization mode: Negative (CYA/AMD); Positive (MEL/AMN)	87.8-97.5 %	LOD: 0.0009-0.011<br>LOQ: 0.003-0.039	[3]
$\sum_4 MEL$<br>MEL, CYA, AMN, AMD	1 mL	None	Add 100 ng of IS mixture, add 3.9 mL of ACN and centrifuge, and divide the supernatant into two: take 1 mL for MEL, AMN, and AMD, add 2 mL of 0.1 mol/L HCl, and add them to MCX	LLE<br>Urine sample mixed with ACN (v/v, 1/3.9), supernatant separated.<br>SPE<br>Measurement of MEL, AMN, AMD: eluted with 3 mL of 5% (v/v) ammoniated methanol.<br>Measurement of CYA: eluted with 3 mL of 2%	UPLC-MS/MS<br>LC column: Water ACQUITY UPLC BEH Amide column (1.7 μm, 2.1 × 100 mm; USA)<br>Mobile phase: 5 mM AmFm (pH 4.0) and ACN<br>Injection volume: 2 μL<br>Ionization mode: Negative (CYA/AMD); Positive (MEL/AMN)	94.6-106.5 %	LOD: 0.03-0.05<br>LOQ: 0.11-0.15	[4]

			solid-phase extraction column.<br>Take 1 mL for CYA, add 2 mL of 2% (v/v) NH_4OH, and add them to MAX SPE.	(v/v) formic acid in methanol.<br>Re-solubilization of 0.5 mL ACN/5 mM AmFm (pH 4.0, v/v, 9/1).				
$\sum_4 MEL$<br>MEL, CYA, AMN, AMD	Two 250 μL aliquots per sample	None	Acidified with 50 μL of 1 % (v/v) FA and alkalized with 5 % (v/v) NH4OH, respectively.<br>A total of 5.0 ng of the IS mixture was added separately.	LLE<br>Acidified/alkalized samples were extracted twice with 2 mL EtAc/IPA (v/v, 95/5);<br>ACN/5 mM AmFm (pH 4.0, v/v, 9:1) was re-solubilized.	HPLC-MS/MS<br>LC column: Luna HILIC column (100 mm × 3.0 mm, 3.0 μm particle size; Phenomenex, Torrance, CA)<br>Guard column: Betasil C18 guard column (20 mm × 2.1 mm, 5 μm particle size; Thermo Scientific, Waltham, MA)<br>Mobile phase: ACN and 5 mM AmFm (pH 4.0)<br>Ionization mode: Negative (CYA/AMD); Positive (MEL/AMN)	74-96 %	LOQ:<br>MEL: 0.20<br>CYA: 0.12<br>AMN: 0.10<br>AMD: -	[5]

Table S2. Analytical methods for the determination of OPEs and their metabolites in human urine.

Analyte	Urine volume	Enzyme Type	Sample preparation	Sample extraction	Instrument method	Recovery rate (%)	LOD/LOQ (ng/mL)	Ref.
OPE metabolites								
$\sum_4 OPE\ metabolites$ DPHP, DNBP, DIBP, BDCIPP	0.5 mL	β-glucuronidase/sulfatase	Deuterated IS mixture with 1 ng OPE added; Buffer: 1 mL 10 mM ammonium acetate (pH 5)	SPE: Sequential passes: 2mL 5% (v/v) ammonia/MeOH, 2mL MeOH, and 2mL water. Elution: 2x 0. 5mL 5% (v/v) ammonia/methanol; 1mL water wash followed by vacuum drying; 2x 0.5mL 5% (v/v) ammonia/methanol elution; concentrated and dried at 37°C in nitrogen stream; 0.1mL ACN re-solubilized.	HPLC-MS/MS LC column: Kinetex HILIC column (100mm×2.1 mm,2.6um); Guard column: Betasil C18 guard column (20mm×2.1 mm, 5um)	70.4-133%	LOD:0.012-0.044	[6]
$\sum_3 OPE\ metabolites$ DPHP, BDCIPP, DNBP	0.4 mL	β-glucuronidase/sulfatase	-	SPE: 60 mg Strata XAW Polymer Packing (1.5 mL liquid volume, Phenomenex); eluent concentrated and reconstituted.	Reversed-phase high-performance liquid chromatography（Agilent 1290）；isotope dilution-electrospray ionization tandem mass spectrometry（AB Sciex 5500 Qtrap）	-	LOD:0.05-0.16	[7]
$\sum_2 OPE\ metabolites$ DPHP, BDCIPP	0.2 mL	β-glucuronidase/sulfatase	-	SPE: automated off-line solid-phase extraction (60mg Strata XAW SPE packing, 1.5mL liquid volume);3×400uL of 2%(v/v) NH4OH in methanol purge.	isotope dilution high-performance liquid chromatography-tandem mass spectrometry	98%-108%;	LOD:0.1	[8]

$\sum_2 OPE\ metabolites$ DPHP, BDCIPP	5 mL	β-glucoronidase and sulfatase enzymes	Add 1ng deuterium internal standard; buffer: 1M sodium acetate buffer (pH 5); incubation time: 37 °C overnight	Mixed-mode anion-exchange solid-phase extraction; 2% (v/v) NH_4OH/methanol elution; eluent concentrated and redissolved	electrospray ionization liquid chromatography–tandem mass spectrometry (Agilent Technologies Model 6410); LC column: Phenomenex Luna C18 column	-	-	[9]
$\sum_2 OPE\ metabolites$ DPHP, BDCIPP	5 mL	u-glucuronidase and sulfatase enzymes	Add internal standard solution; enzyme volume: 250 uL (1000 units/mL u-glucuronidase and 33 units/mL sulfatase in 0.2 M sodium acetate buffer); buffer: 1.75 mL 1 M sodium acetate (pH 5); incubation time: overnight at 37 °C.	SPE: StrataX-AW (60mg,3ml) column; 500μL of water–methanol (1:1, v/v) reconstituted; $^{13}C_2$-DPHP was added to quantify the recovery of the internal standard.	LC-MS/MS: electrospray ionization (ESI) liquid chromatography tandem mass spectrometry); LC column: Phenomenex Luna C18 column	IS recovery: 34%-98%	LOD:0.003-0.846	[10]
$\sum_2 OPE\ metabolites$ DIBP, DPHP	1 mL		Add 2 ng of internal standard solution; buffer: 1 ml of 10 mmol ammonium acetate buffer (pH=5)	SPE STRATA-X-AW cartridge (60 mg, 3 ml); 2 ml 5% ammonium hydroxide dissolved in MeOH elution; concentrated and re-solubilized after elution.	HPLC-MS/MS LC column: Kinetex HILIC column (100mm×2.1 mm, 2.6um); Mobile phases:(A) HPLC-grade water (10 mM ammonium acetate): methanol (2:3 v/v); (B) ACN	70-113%	LOQ:0.001-0.50	[11]

Reference

1. Zhu, H.; Kannan, K. Inter-day and inter-individual variability in urinary concentrations of melamine and cyanuric acid. *Environment International* 2019, *123*, 375-381, doi:10.1016/j.envint.2018.12.018.
2. Zhang, M.; Li, S.; Yu, C.; Liu, G.; Jia, J.; Lu, C.; He, J.; Ma, Y.; Zhu, J.; Yu, C. Determination of melamine and cyanuric acid in human urine by a liquid chromatography tandem mass spectrometry. *Journal of Chromatography B-Analytical Technologies in the Biomedical and Life Sciences* 2010, *878*, 758-762, doi:10.1016/j.jchromb.2010.01.020.
3. Liu, Y.; Zhang, Q.; Zhao, L.; Hua, L.; Xu, K.; Shi, Y.; Chen, S.; Zhao, H.; Zhu, H.; Wang, S. Unraveling the contribution of melamine tableware for human internal exposure to melamine and its derivatives: Insights from crossover and biomonitoring studies. *The Science of the total environment* 2024, *955*, 176971-176971, doi:10.1016/j.scitotenv.2024.176971.
4. Liu, S.; Dong, R.; Wang, Y.; Yang, Z.; He, G.; Chen, B. Twenty-four-hour temporal trend of melamine and its derivatives in urine in association with meal consumption: a panel study in Shanghai, China. *Environmental Science and Pollution Research* 2023, *30*, 120225-120235, doi:10.1007/s11356-023-30740-0.
5. Zhu, H.; Loganathan, B.G.; Kannan, K. Occurrence and Profiles of Melamine and Cyanuric Acid in Bovine Feed and Urine from China, India, and the United States. *Environ. Sci. Technol.* 2019, *53*, 7029-7035, doi:10.1021/acs.est.9b00469.
6. Hernandez-Castro, I.; Eckel, S.P.; Howe, C.G.; Niu, Z.; Kannan, K.; Robinson, M.; Foley, H.B.; Grubbs, B.; Al-Marayati, L.; Lerner, D.; et al. Sex-specific effects of prenatal organophosphate ester (OPE) metabolite mixtures and adverse infant birth outcomes in the maternal and developmental risks from environmental and social stressors (MADRES) pregnancy cohort. *Environmental Research* 2023, *226*, doi:10.1016/j.envres.2023.115703.
7. Kang, H.; Lee, J.; Lee, J.P.; Choi, K. Urinary metabolites of organophosphate esters (OPEs) are associated with chronic kidney disease in the general US population, NHANES 2013–2014. *Environment International* 2019, *131*, doi:10.1016/j.envint.2019.105034.
8. Yang, W.; Braun, J.M.; Vuong, A.M.; Percy, Z.; Xu, Y.; Xie, C.; Deka, R.; Calafat, A.M.; Ospina, M.; Yolton, K.; et al. Patterns of urinary organophosphate ester metabolite trajectories in children: the HOME Study. *Journal of Exposure Science & Environmental Epidemiology* 2023, *34*, 251-259, doi:10.1038/s41370-023-00605-2.
9. Hammel, S.C.; Zhang, S.; Lorenzo, A.M.; Eichner, B.; Stapleton, H.M.; Hoffman, K. Young infants' exposure to organophosphate esters: Breast milk as a potential source of exposure. *Environment International* 2020, *143*, doi:10.1016/j.envint.2020.106009.
10. Hoffman, K.; Lorenzo, A.; Butt, C.M.; Adair, L.; Herring, A.H.; Stapleton, H.M.; Daniels, J.L. Predictors of urinary flame retardant concentration among pregnant women. *Environment International* 2017, *98*, 96-101, doi:10.1016/j.envint.2016.10.007.
11. Zhang, T.; Bai, X.-y.; Lu, S.-y.; Zhang, B.; Xie, L.; Zheng, H.-c.; Jiang, Y.-c.; Zhou, M.-z.; Zhou, Z.-q.; Song, S.-m.; et al. Urinary metabolites of organophosphate flame retardants in China: Health risk from tris(2-chloroethyl) phosphate (TCEP) exposure. *Environment International* 2018, *121*, 1363-1371, doi:10.1016/j.envint.2018.11.006.
